# Empirically Based Psychosocial Therapies for Schizophrenia: The Disconnection between Science and Practice

**DOI:** 10.1155/2013/792769

**Published:** 2013-04-23

**Authors:** Glenn D. Shean

**Affiliations:** College of William & Mary, P.O. Box 8795, Williamsburg, VA 23187-8795, USA

## Abstract

Empirically validated psychosocial therapies for individuals diagnosed with schizophrenia were described in the report of the Schizophrenia Patient Outcomes Research Team (PORT, 2009). The PORT team identified eight psychosocial treatments: assertive community treatment, supported employment, cognitive behavioral therapy, family-based services, token economy, skills training, psychosocial interventions for alcohol and substance use disorders, and psychosocial interventions for weight management. PORT listings of empirically validated psychosocial therapies provide a useful template for the design of effective recovery-oriented mental health care systems. Unfortunately, surveys indicate that PORT listings have not been implemented in clinical settings. Obstacles to the implementation of PORT psychosocial therapy listings and suggestions for changes needed to foster implementation are discussed. Limitations of PORT therapy listings that are based on therapy outcome efficacy studies are discussed, and cross-cultural and course and outcome studies of correlates of recovery are summarized.

## 1. Introduction

Schizophrenia is a group of disorders in which biological, psychological, and sociocultural factors interact synergistically during all phases of the disorder to result in impairments in interpersonal, practical life skills, and vocational functioning. In order to ameliorate the range of symptoms and functional impairments associated with this diagnosis, comprehensive treatment programs are necessary that provide an array of continuing services, including medication management, access to appropriate psychosocial therapies, and assistance with housing, employment, and sources of financial sustenance. Antipsychotic medications can be effective in reducing symptoms and risk of relapse; however, many individuals continue to evidence significant functional and social deficits after acute symptoms have been ameliorated. Growing concerns about the recurring nature of the disorder as well as the severity of functional psychosocial deficits have contributed to an increased emphasis on the importance of empirically validated psychosocial therapies that foster recovery, beyond symptom remission [1]. Recovery-oriented psychosocial treatment programs ideally are designed to provide services designed to help participants learn how to more effectively live with vulnerabilities, reduce interpersonal and social deficits, and promote improved social adaptation and general life functioning [2]. Progress in achieving recovery is fostered by access to comprehensive mental health treatment programs that offer an array of services including access to pharmacological and psychosocial treatments designed to reduce symptoms and enhance general life functioning [3].

In 1992, the Agency for Health Care Policy and Research and the US National Institute of Mental Health funded the Schizophrenia Patient Outcomes Research Team (PORT) to develop and disseminate recommendations for the treatment of schizophrenia based on existing scientific evidence. PORT recommendations, first published in 1998, revised in 2003, and most recently issued in 2009, have played an important role in the dissemination of guidelines for providing current evidence-based practices for schizophrenia [4]. The most recent PORT psychosocial therapy recommendations include 13 recommendations for psychopharmacological and 8 psychosocial treatments for schizophrenia [5]. The most recent PORT committee states that “currently available treatment technologies, when appropriately applied and accessible, should provide the vast majority of patients with significant relief from psychotic symptoms and improved opportunities to lead more fulfilling lives in the community” [2, page 193].

PORT recommendations have been effective in spreading the word that there are psychosocial treatments that work for serious mental disorders, including schizophrenia; unfortunately, these recommendations have not been widely implemented. The lack of effective implementation of PORT recommendations was recently acknowledged by the US President's New Freedom Commission that concluded “the US mental health system is in shambles, and incapable of delivering and financing effective treatments” [6]. The President's commission called for major reforms to revolutionize mental health care for schizophrenia in the US. This paper summarizes the 2009 PORT psychosocial therapy recommendations and discusses a variety of issues and obstacles to implementation of the PORT recommendations. Additional research relevant to fostering the goals recovery but, not derived from randomized control efficacy therapy studies, is described and the implications of these studies for treatment, programming, and research are discussed.

## 2. PORT Psychosocial Therapy Recommendations

### 2.1. Assertive Community Treatment

The PORT committee recommends that systems serving persons with schizophrenia should include a program of assertive community treatment (ACT). ACT programs should be provided in particular for those individuals who are at high risk for repeated hospitalizations or have recent homelessness. The key elements of ACT include a multidisciplinary team including a medication prescriber, a shared caseload among team members, direct service provision by team members, a high frequency of patient contact, low patient-to-staff ratios, and outreach to patients in the community. ACT is not a specific therapeutic strategy so much as a way or organizing services to more effectively integrate individuals with severe mental illness into life in the community. ACT programs are designed and intended to improve coordination, integration, and continuity of services among providers over an extended period of time and have been demonstrated to be effective in decreasing symptom severity, improving medication compliance, reducing hospitalizations and capitated costs, and improving satisfaction among both patients and families [4, 7]. When ACT programs are implemented with high fidelity to the model, this approach is successful in reducing homelessness and improving housing stability [8]. ACT programs have also been demonstrated to be effective in promoting client choice, enhancing a recovery perspective, and enhancing meaningful community integration [8]. It is important to note that ACT programs can and should be integrated with and offered as part of an array of psychosocial treatment approaches, including supported employment, skills training, and substance abuse treatment programs [9–11].

### 2.2. Supported Employment

The PORT committee recommends that any person with schizophrenia who has the goal of employment should be offered supported employment (SE) services to assist them in obtaining and maintaining competitive employment. The key elements of effective SE programs include individually tailored job development, rapid job search, availability of ongoing job supports, and integration with existing vocational and mental health services. Surveys indicate that about 60% percent of people diagnosed with serious mental illnesses are capable of employment, and 70% say they would like to be working, but fewer than 15% were employed even temporarily, and less than 25% receive any form of vocational assistance [12]. Several intrinsic and extrinsic factors make it difficult for individuals diagnosed with schizophrenia to find and maintain suitable employment. Intrinsic factors include increased vulnerability to stress, the episodic nature of the disorder, possible neurocognitive deficits, the presence of idiosyncratic behaviors and beliefs, social anxieties, low self-efficacy beliefs, and lack of vocational and social skills. Extrinsic factors include stigma on the part of potential employers, hiring practices that automatically eliminate applicants with spotty employment histories, government disability programs that discourage employment, and lack of access to appropriate SE services [13]. Evidence indicates that SE services are most effective when combined with additional services including access to medication and an array of psychosocial therapies [14]. Short-term employment rates are significantly improved over traditional vocational placement approaches, and programs that integrate SE with cognitive remediation, skills training, and cognitive behavioral therapy are underway to improve long-term results [4]. While controlled studies indicate that SE programs are effective, it is important to recognize that many participants do not achieve full-time employment so that of the approximately one-half of all individuals diagnosed with schizophrenia who enter SE services, only about 30 percent may transition to active phase of treatment [15]. Of those SE participants placed in competitive employment, most work less than 40 hours a week with earnings below subsistence levels [15]. The effectiveness of SE is related to the fidelity of program implementation [16].

### 2.3. Skills Training

The PORT commission recommends that patients with deficits in the skills needed for everyday activities should be offered opportunities to participate in skills training in order to improve social interactions and other skills needed for independent living. Skills training programs vary, but key elements include behaviorally based instruction, role modeling, rehearsal, corrective feedback, and positive reinforcement [17]. Clinic-based training sessions should be supplemented with opportunities for practice in applying skills in the day-to-day environment [4]. There is a substantial body of evidence that indicates that persons diagnosed with schizophrenia are capable of learning interpersonal and everyday living skills when provided with structured behavioral training that focuses on clearly defined activities, situations, and problems [4]. Skills training results in significant effects on proximal measures of skills; however, evidence is less clear regarding long-term effects and the indirect effects of skills training on ratings of psychopathology or relapse rates. Programs that facilitate the application of skills in everyday environments are more likely to generalize to other settings relevant to the everyday lives of patients living in the community [17]. A meta-analysis of 22 studies including 1,521 participants in randomized controlled trials of social skills training concluded that results indicated a large effect size for content mastery exams, moderate effects for performance-based measures of social and daily living skills, community functioning and negative symptoms, and small effect sizes for other symptoms and relapse rates [18]. Skills training should be implemented in the context of a multielement treatment program that includes medication management, intensive case management, crisis services, family psychoeducation, SE job coaching and training, and access to supported housing [4]. There are several questions and avenues for further study of skills training. First, it is not clear to what degree individuals with better premorbid skill levels and later onset of illness benefit from standard social skills training, as compared those with earlier onset and poor premorbid adjustment, nor is the impact of the presence of neurocognitive deficits on the effectiveness of skills training well understood [4].

### 2.4. Cognitive Behavior Therapy

The PORT committee recommends that persons diagnosed with schizophrenia with persistent psychotic symptoms while receiving adequate pharmacotherapy may benefit from adjunctive cognitively oriented psychotherapy, individually or in group format for 4–9 months, to reduce severity of symptoms. Cognitive behaviorally oriented psychotherapy (CBT) attempts to reduce certain symptoms and enhance functioning by entering into a dialogue that provides rational alternative perspectives to the patient's experiences, with the goal of helping the individual to better understand and cope with issues and experiences that are especially problematic for the individual. The key elements of CBT include collaborative identification of target problems and the development of specific cognitive and behavioral strategies to cope with these problems. Common CBT goals and strategies include helping individuals recognize delusional thoughts, early signs of relapse and learning stress reduction tools and coping strategies. CBT focuses on encouraging individuals to reappraise delusional beliefs in order to reduce distress, reduce negative schemas, more effectively manage stressful environments, change reasoning biases by the application of therapy-assisted disconfirmation strategies, and detailed consideration of the full range of evidence. Studies indicate that CBT can be effective in ameliorating positive symptoms such as delusions and hallucinations and improving social functioning, although its effects are modest [19–21]; however, CBT has not been found to be consistently effective in targeting negative symptoms [4].

### 2.5. Token Economy Interventions

PORT recommends that long-term inpatient or residential care systems for schizophrenic patients are appropriate for token economy behavioral interventions based on positive reinforcement and social learning principles structured to improve personal hygiene, social interactions, and other basic adaptive behaviors. The key elements of token programs are contingent positive reinforcement procedures using tokens that target clearly defined behaviors, using an individualized treatment approach, and the avoidance of punishing consequences. To be effective, token economies should be delivered in the context of safe environments that provide access to basic amenities, evidence-based pharmacological treatment, and the full range of other validated psychosocial interventions. Several advantages accrue from the use of tokens to provide immediate reinforcement of prosocial skills, opportunities to reinforce complex behavioral sequences in an incremental manner, and the opportunity to maintain the reinforcing properties of tokens through access to an array of backup rewards [22]. Effective implementation of token economies can increase in-hospital adaptive behaviors of patients diagnosed with schizophrenia in hospital and residential treatment environments, particularly in cases where residents are socially withdrawn, unmotivated, and have difficulty with performing routine activities of daily living [23] When intensive behavioral treatments, that include token reinforcement, are combined with appropriate pharmacotherapy, the number of patients considered to be treatment refractory is lower than when medication alone or medication plus standard treatment are used [24], so that many withdrawn “institutionalized” patients can be reactivated with consistent external cueing and opportunities for contingent reinforcement [25]. Research is needed to determine the specific benefits of token reinforcement programs for reliably identified subgroups and when administered in combination with other pharmacological and psychosocial treatments such as social skills, SE, and cognitive rehabilitation [4].

### 2.6. Family-Based Services

The PORT commission recommends that persons with schizophrenia who have ongoing contact with their families, including relatives and significant others, should be offered a family intervention that lasts 6–9 months. Family interventions shorter than 6 months but at least 4 sessions in length should be offered to patients who have ongoing contact with families, including relatives and significant others and for whom longer intervention is not feasible. Briefer interventions should include at a minimum education, training, and support. Key elements of family interventions include illness education, crisis intervention, emotional support, and training in how to cope with illness symptoms and related problems. The goals of family-based services are to increase understanding of the disorder, reduce levels of expressed emotion, reduce feelings of isolation, stress, and burden of family members, foster development of coping skills, and develop an ongoing collaborative relationship between family and clinicians. Implementation of family interventions should be guided by collaborative decision making among the patient, family and clinicians. Meta-analyses indicate that family-based interventions help increase medication compliance, reduce symptoms and rehospitalization rates, improve functional and vocational status and perceived stress among patients. Positive family outcomes include decrease family burden and increased satisfaction with family relationships [26–28]. An issue to be addressed is the high attrition rates of key relatives reported in the studies of FE [29]. In the US it is estimated that only about 10% of families of patients diagnosed with schizophrenia receive any form of psychoeducational interventions; in Western Europe the percentage of families receiving the services is only slightly higher at 15% [30]. Barriers to implementing family interventions include (1) structural problems in the mental health system which lead to many patients being placed in nursing homes and prisons rather than participating in community-based programs; (2) failure of clinicians, perhaps due to inadequate training and skills, to implement EST-based family interventions; (3) difficulties in developing an effective therapeutic alliance with many schizophrenic patients, who often lack insight into their illness and reject the need for treatment; (4) frustration of family members who may be overwhelmed by responsibilities caring for their affected family member and reject family-based treatments when offered [30].

### 2.7. Psychosocial Interventions for Alcohol and Substance Use Disorders

Persons with comorbid alcohol or drug use disorders and schizophrenia should be offered access to substance abuse treatment, the key elements of which include motivational enhancement, behavioral strategies that focus on engagement in treatment, coping skills, relapse prevention training, and integration with a broad mental health care model [4]. Studies indicate that integrated treatment increases the likelihood that individuals will stay in treatment with better participation, evidence reductions in substance use, and have fewer hospitalizations and arrests than controls [4]. Integrated treatment mental health and substance abuse treatments have been found to have equivalent effectiveness across traditional clinical case management and ACT clinical formats [31, 32]. 

### 2.8. Psychosocial Interventions for Weight Management and Smoking Cessation

Attention has recently been focused on the poor health status of individuals diagnosed with schizophrenia, including high rates of cardiovascular disease, diabetes, obesity, and reduced life expectancy [33]. The PORT committee recommends that individuals who are overweight (BMI = 25.0–29.9) or obese (BMI > 30.0) should be offered access to a weight loss intervention program at least 3 months in length that includes psychoeducation, focused on nutritional counseling, portion control and caloric expenditure, behavioral self-management including motivational enhancement, goal setting, regular weigh-ins, self-monitoring of food intake and activity levels, along with dietary and physical activity modifications [4] Randomized controlled studies report modest weight loss for overweight participants [32–34]. Those who smoke should be offered access to psychosocial programs to reduce tobacco use. Smoking quit rates among persons diagnosed with schizophrenia in response to psychosocial treatment programs have been modest; nevertheless, the PORT committee recommends access to cessation programs and weight reduction programs due to the high prevalence and associated life-threatening conditions associated with these problems that contribute to poor outcomes and disability [35].

## 3. Summary Statements

Several interventions were recognized by the PORT commission as of potential value but for which the evidence available was not sufficient to merit a treatment recommendation at this time.

### 3.1. Cognitive Rehabilitation

Cognitive impairments among individuals diagnosed with schizophrenia are well documented and account for significant variations in functional disability; in fact cognitive impairment has been found to be more highly correlated with functional impairments in living skills, social competence, employment status, and everyday functioning than clinical symptoms [36–39]. Cognitive rehabilitation refers to efforts that target specific functions such as memory, attention, and reasoning with the goal of improving overall functioning. At present, the available literature on cognitive rehabilitation effectiveness is promising but limited by a lack of evidence of the impact of these programs on broad indicators of psychosocial functioning, wide variation in remediation models, and mixed support in clinical trials [4].

### 3.2. Peer Support and Peer-Delivered Services

Involvement of consumers in planning, delivery, and evaluation of services is recognized as essential to recovery-oriented systems of care for persons diagnosed with schizophrenia. A central tenet of the recovery model is empowerment of the user is important in achieving good outcome. Empowerment helps people reduce their sense of stigma and helps them develop insight into their problems. Consumer involvement can also help to reduce stigma and hiring barriers and provide role models and access to shared experiences that can foster recovery; however, the program effectiveness literature is lacking in numbers of studies conducted with adequate experimental designs to meet recommendation as an empirically established psychosocial treatment approach. Peer-developed and -led programs including psychosocial clubhouse models can help empower patients and enhance prospects of recovery [40, 41]. Questions remain to be answered about selection and training of consumers, and types of services that are most appropriate need to be addressed [4].

### 3.3. Interventions to Increase Adherence to Antipsychotic Medication

Lack of adherence to medication regimens remains a widespread problem and is associated with increased probability of relapse, and hospitalization. Behavioral approaches to increase medication adherence show promise but there is not sufficient evidence from well-controlled studies to merit a recommendation at this time [4].

### 3.4. Recent-Onset Schizophrenia

Misdiagnosis and delay in treatment after initial onset of symptoms is common among individuals eventually diagnosed with schizophrenia and contribute to long-term disability. Packages of treatment approaches (e.g., CBT, family interventions, and supported employment programs) for individuals with recent-onset schizophrenia have been developed that show considerable promise; however, the research literature is judged insufficient to support recommendations at this time [4].

## 4. Limits of Efficacy Studies

PORT committee recommendations are based on the results of psychotherapy efficacy studies. The treatment, no-treatment control group design of therapy efficacy studies that are the basis for PORT recommendations often do not take into account a range of patient characteristics, life history and demographic variables that impact prospects for both remission and recovery. There is a large body of published research, for example, that indicates contributors to course and outcome of schizophrenia including acute onset, educational attainment, prominent affective features, good premorbid functioning, precipitating events, married, female, insight, short duration of untreated psychosis, and no family history of schizophrenia or mood disorder [42–45]. Poor prognosis is associated with insidious onset, asocial premorbid personality, social withdrawal, never married, and positive family history for schizophrenia [42–46]. The relationship between these patient characteristics and psychosocial therapy efficacy has not been adequately studied. Long-term studies are also needed to evaluate the effects of different therapies and combinations of services on the trajectory of recovery of different symptom patterns (e.g., disorganized, reality distortion, and negative symptoms).

## 5. Remission or Recovery

The Remission in Schizophrenia Working Group proposed a set of consensus criteria based on DSM-IV criteria [1]. Remission is described as a state in which patients have experienced an improvement in core symptoms, to the extent that these symptoms no longer interfere significantly with daily life. The Working Group recommended remission criteria consisting of two elements: a symptom-based criterion and a time criterion (duration of 6 months). The symptom criteria include three core symptom dimensions: “reality distortion” (delusions, unusual thought content, hallucinatory behavior), “disorganization” (conceptual disorganization, mannerisms, and posturing), and “negative symptoms” (flat affect, social withdrawal, and lack of spontaneity). Symptom ratings based on these dimensions and duration criteria are recommended as standardized indicators of remission that are necessary to allow for comparisons across studies. The concept of recovery overlaps with remission criteria to a degree, but recovery suggests the application of a long-term perspective and evaluation in terms of broad indicators of cognitive, occupational, emotional functioning, and quality of life [47, 48]. There is ample evidence that provision of opportunities for involvement in functional roles such as employment can have beneficial effects on long-term prospects for recovery [49]. Yet, fewer than 15% of individuals diagnosed with schizophrenia and living in the community in the US are employed in any capacity [50]. The US employment rate contrasts markedly with several other industrialized countries that have adopted policies designed to eliminate obstacles to ex-patients finding work. In Bologna, Italy, for example, nearly half of all ex-patients were employed continuously and more than one-fifth were working full time. In Verona, Italy, nearly 60 percent of schizophrenic patients living in the community were employed, one-quarter full time [50]. The availability of supported employment programs, innovative work organizations, and changes in policies that determine how disability pensions are regulated can foster recovery [49]. Many former patients could benefit from access to partial wage subsidies that encourage them to engage in training and opportunities for graded levels of participation in meaningful work. In parts of Italy where there are fewer policy related disincentives to work, business consortiums have been formed and have successfully employed a mixed workforce of mentally disabled and nondisabled workers to run businesses as varied as hotels, cafés, renovation companies, transport businesses, furniture workshops, cleaning businesses, plant nurseries and work as nursing home aides [50]. There is a need for research on the impact of programs that provide temporary or permanent wage subsidies, training, and incentives to develop innovative rehabilitation models designed to encourage and support enterprises that can harness the productive potential of former patients.

### 5.1. Culture

PORT commission recommendations are based on efficacy studies of psychosocial therapies and do not incorporate evidence from cross-cultural studies, in particular the studies sponsored by the World Health Organization, known as the International Pilot Study of Schizophrenia (IPSS). Results of the IPSS studies have consistently indicated that long-term course and outcome of schizophrenia varies between cultures, with former patients in “developing” countries evidencing significantly better outcome and recovery rates than those living in “developed” countries [51–55]. The IPSS studies were not designed to address the reasons for these differences; however, several factors have been suggested as contributors: (1) less social stigma associated with symptoms of mental illness, (2) the support provided by availability of cohesive and extended supportive social systems, including extended families and tribal groups, and (3) the availability of opportunities for graded resumption of involvement in useful social roles and productive work [50]. The IPSS studies both support and supplement the PORT committee psychosocial therapy recommendations.

## 6. Criminalization of the Mentally Ill

A consequence of lack of access to effective services in many communities is that prisons and jails are serving as repositories for many patients who could otherwise benefit from PORT recommended therapies if they were available. A recent US survey indicated that 31% of females admitted to jail and 14.5% of males had a serious mental illness, and approximately 20% were homeless in the months before their incarceration [56]. In addition to access to psychosocial therapy programs, court-related diversion programs are needed to provide alternatives to incarceration. In order to be maximally effective, diversion programs must be linked to community treatment programs that provide access to an array services such as supported housing and supported employment opportunities, especially for cases involving less serious offenses. Diversion programs during the pretrial detention period based on models such as the “sequential intercept model” have been demonstrated to be effective in reducing the rate of incarceration of the mentally ill [57]. Prison-based programs are also needed for the mentally ill convicted of more serious offenses, in the form of prerelease assistance in making arrangements for housing, entitlements, and links to appropriate treatment agencies are needed to ease reentry back into the community.

## 7. Parallel Universes

For more than a decade after the issuance of the initial PORT psychosocial therapy recommendations, one cannot point to any examples of the comprehensive implementation of these treatment programs. The apparent disconnection between science and practice was documented in the results of a survey conducted by the National Alliance for the Mentally Ill (NAMI). The NAMI survey indicated that more than half of all individuals diagnosed with schizophrenia in the US are impoverished and dependent on public programs for health insurance and income, more than two-thirds are without any form of employment, the most continue to experience repeated crises with one-half being hospitalized in the last year alone; and 40 percent have been arrested because of mental health symptoms [6]. Of the one out of five individuals that received employment-related services in the past year, half rated the quality of the service as poor or fair [6]. A second NAMI survey indicated that while most states in the US have implemented one or more of the psychosocial ESTs, the vast majority of individuals who can benefit from these programs were not receiving them [58]. Several state agencies have developed tool kits to guide implementation of PORT guidelines, but these efforts have been inadequate and underresourced [59–62].

There are many reasons for the disconnection between science and practice; among these a lack of funding and confused lines of authority and organization for planning, budgeting, prioritizing, and implementing services are prominent. The consequences of organizational disarray include lack of access to skilled mentoring and monitoring, inadequate training, inadequate assessment, uncoordinated planning, and lack of financial provisions [63, 64]. Government policies and the manner in which they are implemented often support practices that foster institutional care and incarceration, rather than providing incentives and support for the implementation of comprehensive programs community-based treatment programs. We know a fair amount about what works in the treatment of schizophrenia. The problem today is not the lack of knowledge although much remains to be learned. The task of fostering recovery is not straightforward or easy as many individuals diagnosed with schizophrenia have significant cognitive and social impairments that impair their motivation for change. On the other hand, we will never know an individual's potential for recovery that individual is provided appropriate services and opportunities. The PORT committee has identified a number of empirically supported psychosocial treatments that can help counteract and defuse the impact of schizophrenia psychosis and foster recovery. These recommendations are an important beginning, but the most critical tasks ahead involve gaining access to resources, policy changes, and improved organizational structures needed for effective implementation and support for PORT recommendations.

## 8. Conclusion

Clinical services for individuals diagnosed with schizophrenia can be provided to foster recovery when the following minimal requirements are met: (1) treatment of the acute phase of the disorder in small, noncoercive settings; (2) provision of access to a range of independent and supervised, noninstitutional accommodations; (3) programs and support for the care offered by the families of persons with schizophrenia if readily available; (4) a range of opportunities to participate in a variety of graded work are offered that are tailored to be neither too demeaning or too stressful; (5) economic incentives to work are made available, including wage subsidies; (6) pathways to economic and social advancement are made available through cooperative businesses, housing, and services; (7) the rights of people and their families as fully integrated and respected members of society are fully respected; and (8) administration of antipsychotic drugs is viewed as a supplement to psychosocial therapies, not as a substitute for them [50].
